# Amelioration of Photoreceptor Degeneration by Intravitreal Transplantation of Retinal Progenitor Cells in Rats

**DOI:** 10.3390/ijms25158060

**Published:** 2024-07-24

**Authors:** Jing Yang, Geoffrey P. Lewis, Chin-Hui Hsiang, Steven Menges, Gabriel Luna, William Cho, Nikolay Turovets, Steven K. Fisher, Henry Klassen

**Affiliations:** 1 Gavin Herbert Eye Institute, Sue & Bill Gross Stem Cell Research Center, University of California, Irvine, CA 92697, USA; 2 Neuroscience Research Institute, University of California, Santa Barbara, CA 93106, USA

**Keywords:** stem cells, retinal dystrophy, neuroprotection, electroretinogram, intraocular injection

## Abstract

Photoreceptor degeneration is a major cause of untreatable blindness worldwide and has recently been targeted by emerging technologies, including cell- and gene-based therapies. Cell types of neural lineage have shown promise for replacing either photoreceptors or retinal pigment epithelial cells following delivery to the subretinal space, while cells of bone marrow lineage have been tested for retinal trophic effects following delivery to the vitreous cavity. Here we explore an alternate approach in which cells from the immature neural retinal are delivered to the vitreous cavity with the goal of providing trophic support for degenerating photoreceptors. Rat and human retinal progenitor cells were transplanted to the vitreous of rats with a well-studied photoreceptor dystrophy, resulting in substantial anatomical preservation and functional rescue of vision. This work provides scientific proof-of-principle for a novel therapeutic approach to photoreceptor degeneration that is currently being evaluated in clinical trials.

## 1. Introduction

Photoreceptor cells do not regenerate in the mammalian retina, and therefore the loss of these highly specialized neurons results in irreversible visual deficits. In humans, the dysfunction and death of rod and cone photoreceptors underlies many untreatable forms of blindness, notably including degenerative diseases such as age-related macular degeneration (AMD) and retinitis pigmentosa (RP) [1,2,3]. Despite recent success treating AMD-associated choroidal neovascularization [4], the lack of a method to prevent photoreceptor loss continues to hinder efforts to address diseases of this type. Multiple groups have endeavored to solve this problem either through cell replacement or delivery of neurotrophic factors to the diseased microenvironment, and a number of cell-based therapeutic programs have begun clinical testing [5]. Of these, efforts utilizing subretinal transplantation of pluripotent stem cell-derived retinal pigment epithelium (RPE) into patients with AMD have attracted particular attention. This approach aims to promote photoreceptor survival and function through replacement of diseased RPE underlying the macular region of the retina [6,7,8,9,10,11] (). Alternatively, multipotent neural progenitor cells derived from brain or neural retinal tissue have been placed under the macula in either AMD [12] or RP [13] patients, respectively. Other groups have taken the relatively simpler approach of injecting various cell types of extra-neural origin, most typically bone marrow-derived (e.g., mesenchymal cells or CD34+ hematopoietic progenitors), into the vitreous cavity [14,15] or beneath the retina (NCT00458575; NCT01226628), with the goal of eliciting a neurotrophic response.

Clinically, cell-based strategies continue to face challenges, including potential side effects related to product delivery such as perforation and focal detachment of a frail, degenerating retina, achieving meaningful levels of donor cell integration and cell replacement, or sustained delivery of neurotrophic agents. Uncertainties also surround the use of non-ocular donor cells in the eye, including concerns over safety [15] and whether a reliable benefit can be achieved [16]. What has not been explored is intravitreal delivery of neural cell types for neuroprotection rather than cell replacement. Such an approach could combine the biological advantages of a homologous cell type with an expedient, less invasive delivery method. Here we show that allogeneic retinal progenitor cells (RPCs) induce photoreceptor rescue when transplanted to the vitreous cavity of the dystrophic Royal College of Surgeons (RCS) rat. These initial findings are then extended to human RPCs using the same model, thus providing preclinical proof-of-concept for the application of this approach in patients with photoreceptor degeneration.

## 2. Results

**Figure 1:**
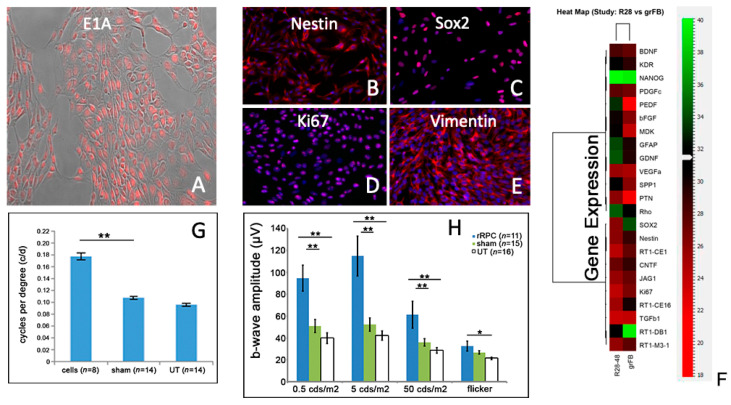
Allogeneic RPCs ameliorate degeneration in RCS rats: donor cells and host function. (**A**–**E**) Rat-derived RPCs labeled for E1A reporter gene (red) (**A**), nestin (**B**), sox2 (**C**), Ki-67 (**D**), and vimentin (**E**) (DAPI = blue). (**F**) Relative expression by rRPCs of selected genes of interest, including cytokines, as compared to allogeneic fibroblasts. (**G**,**H**) Functional performance of rRPC- versus sham- and untreated eyes in dark-eyed dystrophic RCS recipients as assessed via optomotor (**G**) and ERG (**H**) testing (cell = rRPC, sham = saline, UT = untreated, * = *p* < 0.05, ** = *p* < 0.01).

**Figure 2:**
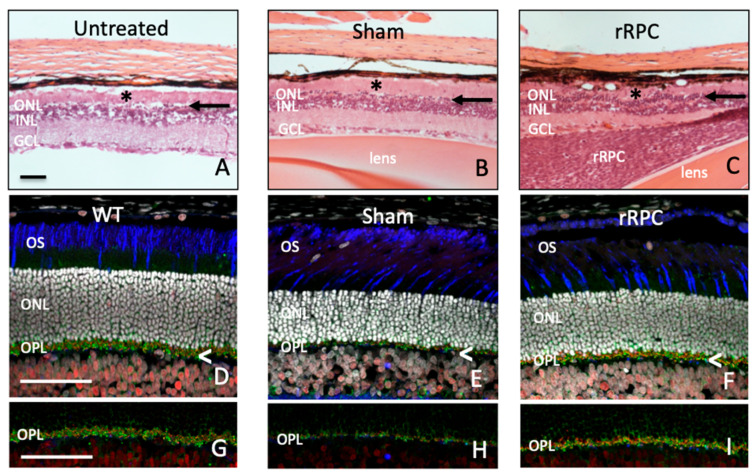
Allogeneic RPCs ameliorate degeneration in RCS rats: host anatomy. Assessment of photoreceptor cell loss via relative attenuation of the outer nuclear layer (ONL), comparing untreated (**A**), sham- (**B**), and rRPC-treated (**C**) retinas at 3 months of age. Note: the residual ONL is thin compared to the adjacent INL and indicated with lettering (ONL) as well as arrows (black); * = subretinal debris zone. Relative integrity of the outer plexiform layer (OPL) of non-dystrophic wildtype rats (**D**,**G**) as compared to sham- (**E**,**H**) and rRPC-treated (**F**,**I**) dystrophic RCS rats, assessed at an earlier time point (P35) and therefore prior to substantial degeneration of the ONL (bright white nuclei). Synaptophysin (green) and CtbP2 (red) label structures within the OPL, while cone sheaths are labeled with peanut agglutinin (blue), DAPI (white). (**G**–**I**) Cropped versions of above images (**D**–**F**) with only synaptophysin (green) and CtbP2 (red) labeling to better visualize differences in OPL preservation between conditions. Scale bar = 50 µm.

Prior work has shown that neural progenitor cells survive as allografts in the rodent eye, specifically within the vitreous, retina, and subretinal space, without the need for immune suppression [17,18,19]. Our initial goal was to establish an allogeneic animal model using the RCS rat, in which a defect in the MerTK gene results in photoreceptor degeneration [20]. Using an RPC line of rat origin, it was first confirmed that the cells expressed the endogenous E1A reporter gene in culture (Figure 1A), along with the non-specific RPC-associated markers nestin, sox2, vimentin, and Ki-67 (Figure 1B–E). Initial results using qPCR revealed the expression of multiple cytokines that might exert a neurotrophic influence (Figure 1F) on photoreceptor cells. Following injection into the vitreous cavity, the dissociated cells displayed a strong tendency to aggregate, and the resulting grafts were readily visualized histologically. Clusters of transplanted RPCs were most frequently located near the peripheral retina, adjacent to the injection site. Alternatively, grafted cells were found more centrally within the vitreous or, in some cases, adhering to the vitreal surface of the retina or posterior of the lens. Definitive integration of donor cells into the host neural retina was not observed. After survival times of 6–8 weeks, functional testing demonstrated a positive treatment effect for cells when compared to sham- or untreated controls. This was shown first via optomotor testing (Figure 1G) and then via electroretinography (Figure 1H). Subsequent histological analysis revealed significant partial rescue of the outer nuclear layer (ONL) of approximately four rows of photoreceptor nuclei in RPC-treated eyes (Figure 2C), as opposed to one to two rows in sham-treated (Figure 2B) and a single broken row in untreated controls (Figure 2A). Additional evidence of anatomical preservation came from examination of the outer plexiform layer (OPL), where photoreceptor cell bodies make synaptic contact with second-order neurons. Cell-treated eyes showed improved OPL thickness compared to controls (Figure 2D–I), and this finding was evident at earlier time points when the ONL was still relatively intact.

**Figure 3:**
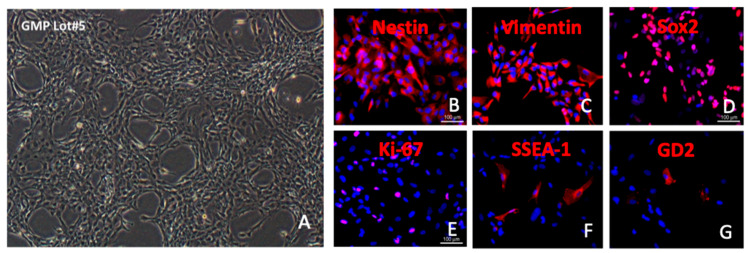
Characteristics of human RPCs in culture. (**A**–**G**) Grown as an adherent monolayer and viewed in phase contrast (**A**); labeled for Nestin (**B**), Vimentin (**C**), Sox2 (**D**), Ki-67 (**E**), SSEA-1/CD-15 (**F**), and GD_2_ ganglioside (**G**); counter-labeled with DAPI (blue). Scale bar = 100 µm.

**Figure 4:**
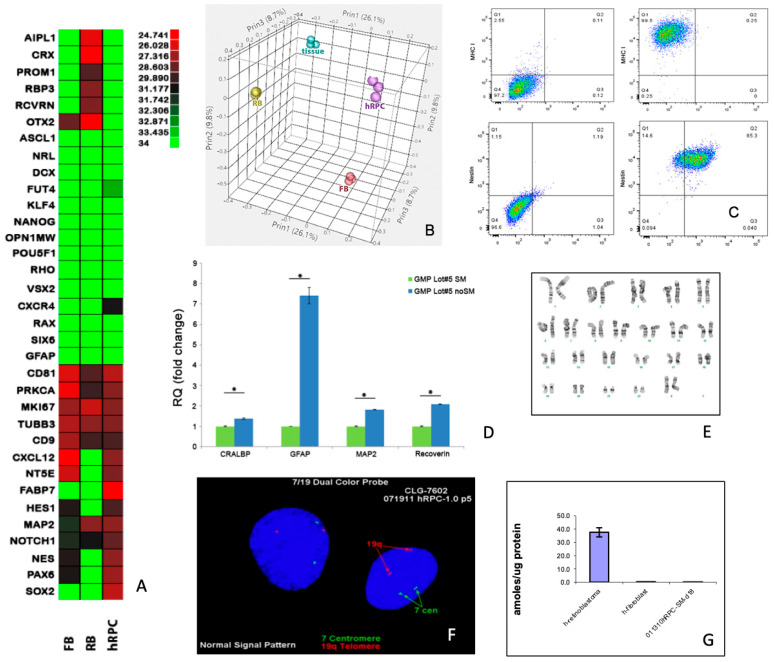

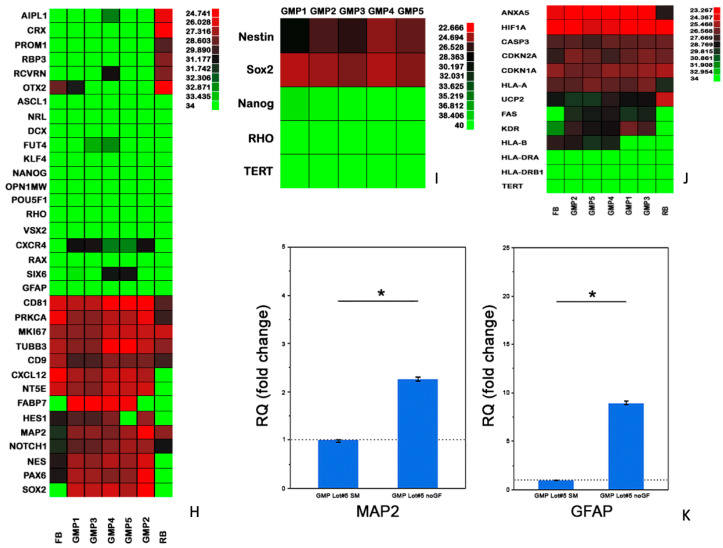
Characteristics of human RPCs: in vitro analysis. (**A**) Expression by hRPCs of selected genes of interest, as compared to syngeneic fibroblasts and an allogeneic retinoblastoma cell line. (**B**) Principal component analysis of the global transcriptome obtained via microarray shows clustering of replicate samples and separation among hRPCs, human fibroblasts (FBs), retinoblastoma cells (RBs), and fetal retinal tissue. (**C**) Flow cytometric analysis comparing expression of MHC class I (vertical axis) versus class II (horizontal) in upper right scatter plot and co-expression of nestin (vertical) and sox2 (horizontal) in lower right scatter plot, with appropriate isotype controls in upper left and lower left plots, respectively. (**D**) Differentiation of hRPCs via growth factor withdrawal (blue bars) induces expression of glial-associated markers CRALBP and GFAP, as well as neuronal markers MAP2 and Recoverin, within the cultured population, versus undifferentiated controls (green bars). (**E**–**G**) Cultured hRPCs have a normal 46, XX karyotype without chromosomal abnormalities (**E**), as confirmed by FISH (**F**), and are negative for telomerase activity, as are syngeneic fibroblasts but not retinoblastoma cells (**G**). (**H**–**K**) Analysis of human RPCs across cell manufacturing lots. Comparison of gene expression levels of 5 different lots (GMP1–5) manufactured for clinical use, using qPCR (**H**–**J**). Also tested were syngeneic human fibroblasts (FB) and a retinoblastoma line (RB). (**K**) Cells from one of the lots (GMP5) were differentiated via growth factor withdrawal (noGF) and evaluated for changes in expression of the markers MAP2 and GFAP relative to undifferentiated controls (SM). Scale bars = 50 µm; * = *p* < 0.05.

The above support for the use of intravitreal RPCs as an allogeneic cell-based treatment for photoreceptor degeneration motivated the development of analogous human RPCs (hRPCs), suitable for use in clinical trials. These hRPCs were derived by the authors under GMP-compatible conditions at the University of California, Davis. In culture, the cells adhered to fibronectin-coated flasks while exhibiting the characteristic morphology (Figure 3A). Immunocytochemistry confirmed expression of the known RPC markers nestin, vimentin, sox2, and Ki-67 (Figure 3B–E), consistent with the rat cells above, as well as SSEA-1 (CD15) and GD2 ganglioside (Figure 3F,G), as previously reported for human RPCs [21]. Microarray analysis showed that the global transcriptome of cultured hRPCs clearly segregated from fetal retinal tissue (i.e., source material), autologous fibroblasts, and an allogeneic retinoblastoma cell line (Figure 4B). qPRC confirmed that RPCs could also be distinguished from these other cells based on differential expression of selected genes (Figure 4A). Flow cytometry was used to evaluate expression of selected surface and cytoplasmic markers. Specifically, the cells were shown to co-express nestin and sox2, as well as class I but not class II MHC antigens (Figure 4C). Because transfer to the microenvironment of the vitreous cavity may result in phenotypic changes in donor cells, growth factor withdrawal was used to examine the baseline propensity towards cellular differentiation in culture. This resulted in differentiation of RPCs along either glial or neuronal lineages, with the former evidenced by elevated expression of GFAP (Figure 4D) in a subset of cells. Low-passage cells maintained a normal karyotype (Figure 4E), confirmed with FISH (Figure 4F), and did not express elevated levels of telomerase, consistent with their status as non-immortal progenitor cells (Figure 4G). Five lots of hRPCs were grown under GMP-like conditions. Comparison between lots showed relatively consistent patterns of marker expression (Figure 4H–J), as well as the ability to differentiate along either neuronal or glial lineages (Figure 4K).

**Figure 5:**
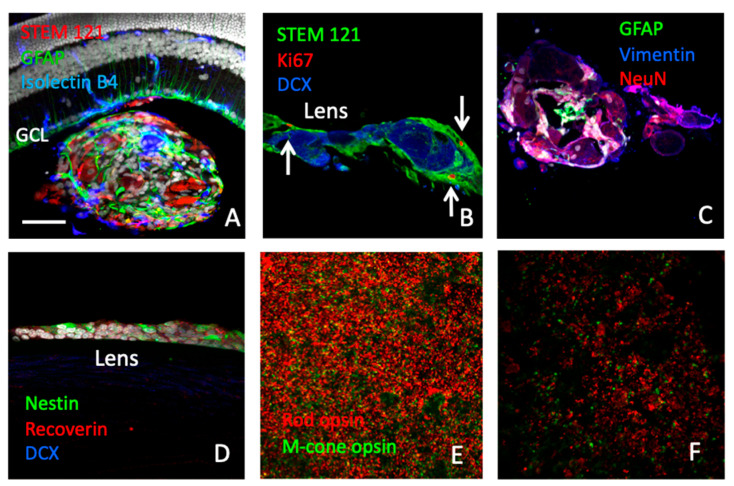
Transplanted hRPCs ameliorate retinal degeneration in RCS rats. (**A**–**D**) Section of rat eye showing human donor cells (red) clustering post injection to form aggregates in the posterior vitreous cavity (**A**,**C**), with occasional adhesion to inner retinal surface but absence of intraretinal hRPC migration. An alternate location for transplanted cells was the posterior lens capsule (**B**,**D**). Donor cells were labeled for Ki-67 (red, arrows) (**B**), Nestin (**D**), Vimentin (**C**), DCX (**B**,**D**), GFAP (**A**,**C**), NeuN (**C**), and Recoverin (**D**). (**E**,**F**) Wholemounts viewed en face at photoreceptor level using confocal microscopy and computer-generated montages showing rhodopsin (red) and middle wavelength cone opsin (green) expressing profiles in hRPC- (**E**) and untreated (**F**) eyes. Scale bar = 50 µm.

**Figure 6:**
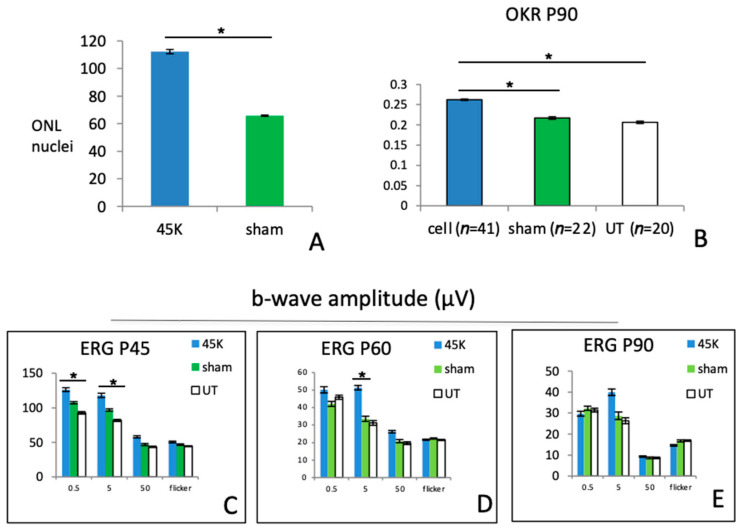
(**A**) Analysis of ONL cell counts from retinal cross-sections of hRPC- and sham-treated retinas. (**B**) Optomotor responses (cycles per degree) comparing sensitivity of hRPC-treated eyes to sham- and untreated (UT) controls. (**C**–**E**) ERG responses in hRPC-treated eyes (45k cell dose) compared to sham- and untreated controls across three in-life time points (P45, P60, P90). * = *p* < 0.05.

We next tested the efficacy of human cells in the RCS rat model. Human-to-rat xenografts (hRPCs) were viable for brief periods (<2 weeks) in the vitreous cavity without immune modulation (Figure 5A); however, the use of an immunosuppressive regimen was necessary to evaluate the potential therapeutic effect at longer time points [22]. With immune suppression, hRPCs survived out to the P90 endpoint (approx. 68 days post-transplantation). Donor cells typically formed spherical cellular aggregates in the vitreous (Figure 5A,C), although they could also be found adhering to surrounding structures, including the retina and posterior lens capsule (Figure 5B,D). Cells within the grafts expressed the retinal progenitor-associated markers nestin, vimentin, and Ki-67, or neuronal or glial lineage markers such as DCX, NeuN, recoverin, or GFAP, indicative of ongoing differentiation (Figure 5B–D). Host RCS retinas showed an hRPC-associated treatment effect, with increased survival of both rods and cones (Figure 5E,F) and quantified as an increased number of nuclear profiles present in the outer nuclear layer (ONL) of hRPC-treated versus sham (*p* > 0.05) (Figure 6A). The visually mediated optomotor response (OR), as measured in awake, unrestrained RCS rats, showed evidence of improved spatial resolution over control animals, both untreated and sham (*p* > 0.05) (Figure 6B). Cell-treated eyes also showed improved ERG responses over a variety of stimulus conditions and range of in-life time points (Figure 6C–E), although overall the results using hRPCs were less pronounced than with the allogeneic rat cells seen above (Figure 1H). There was a general decline in ERG response amplitudes with age and a concomitant decline in the number of stimulus parameters achieving statistical significance, although a signal could still be discerned for hRPC-treated eyes.

**Figure 7:**
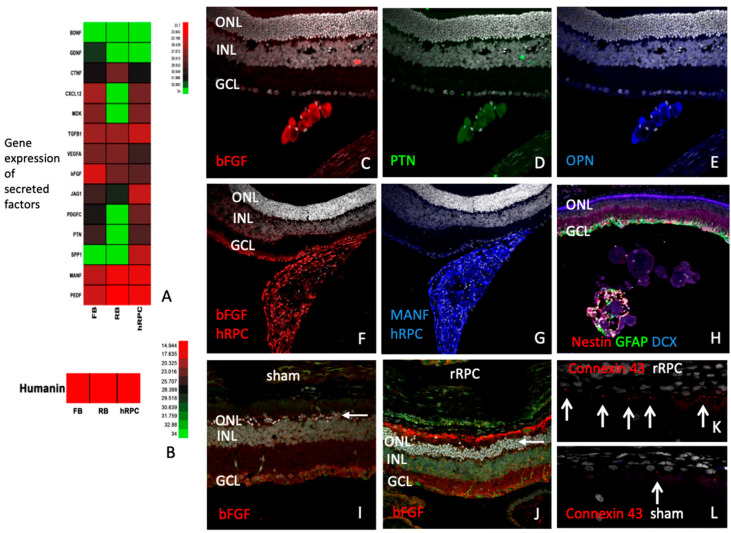
Expression of cytokines and modulation of retinal cell types by RPCs. (**A**,**B**) Relative expression of selected cytokines by cultured hRPCs, measured by qPCR, as compared to retinoblastoma cells (RB) and fibroblasts (FB). (**C**–**E**) Co-expression of candidate cytokines bFGF (**C**), PTN (**D**), and OPN (**E**) within an intravitreal hRPC graft (at P32) and co-expression of bFGF (**F**) and MANF (**G**) by another hRPC graft (at P28), assessed using immunohistochemistry. (**H**) Expression pattern of Nestin, GFAP, and DCX within an intravitreal hRPC graft at a later time point (P90). (**I**,**J**) Relative expression of bFGF in sham- (**I**) and rRPC-treated dystrophic RCS retinas (P90), showing relative labeling within the region of rod outer segments (red), located above the nuclei (bright white, DAPI) of the ONL (arrows). (**K**,**L**) Relative expression of Connexin 43 in sham- (**K**) and rRPC-treated (**L**) dystrophic RCS eyes (P35), seen as fine punctate labeling in the region of the RPE layer (arrows). Scale bar = 50 µm.

To better understand RPC-associated treatment effects, factors produced by the cells were investigated, as well as host cellular responses. Compared to retinoblastoma cells or fibroblasts, one factor preferentially expressed by RPCs was osteopontin (OPN), also known as SPP1 (Figure 7A). Other factors expressed at high levels were PEDF, MANF, JAG1, and TGF beta1. Additional factors expressed by hRPCs included pleiotrophin (PTN), platelet-derived growth factor-C (PDGFC), basic fibroblast growth factor (bFGF), midkine (MDK), and CXCL12. The established neurotrophic factors BDNF and GDNF did not appear to be expressed by hRPCs in culture, whereas the novel cytoprotective candidate humanin was (Figure 7B). Following transplantation to the eye, hRPCs maintained expression of factors, including bFGF, PTN, OPN, and MANF, as demonstrated by IHC (Figure 7C–G). Donor cells were labeled with either immature or lineage markers, indicative of progressive differentiation within the grafts (Figure 7H).

**Figure 8:**
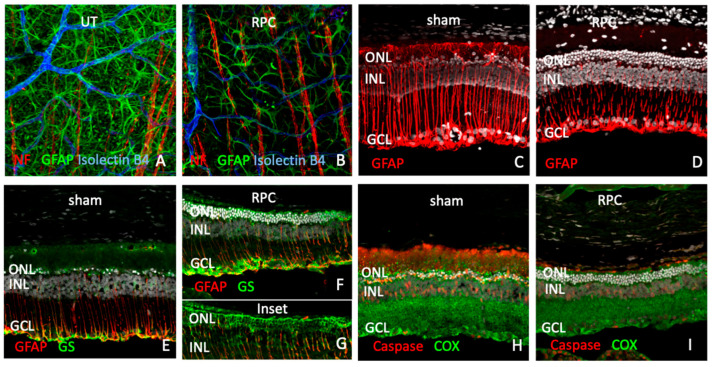
Glial activation patterns in intravitreal RPC-treated dystrophic RCS retinas (at P90). (**A**,**B**) Retinal wholemounts: GFAP (green), Isolectin B4 (blue), Neurofilament (red) in untreated (**A**) and hRPC-treated (**B**) eyes. (**C**,**I**) Retinal cross-sections: GFAP (red), DAPI (white) in sham- (**C**) and rRPC-treated eyes (**D**). Glutamine synthetase (GS, green), GFAP (red), and DAPI (white) in sham- (**E**) and **r**RPC-treated eyes (**F**,**G**). GS labeling better visualized without DAPI (**G**). Caspase 3 (red), cytochrome oxidase (COX, green), and DAPI (white) labeling in sham- (**H**) and rRPC-treated (**I**) eyes. Scale bar = 50 µm.

Examination of host retinas revealed a variety of treatment-related responses among local cell types. Labeling for bFGF was localized to photoreceptors and, more specifically, appeared to correspond to the region of outer segments (Figure 7I,J), as previously reported [23]. The loss of connexin 43 by RPE cells, associated with the dystrophic process, was less pronounced in RPC-treated eyes (Figure 7K,L). The characteristic upregulation of GFAP by hypertrophic Mueller cells and astrocytes in the degenerating RCS retina was also reduced in RPC-treated eyes, as viewed en face via wholemounts (Figure 8A,B) or via cross-sections (Figure 8C–G). Compartmentalization of molecular response within Mueller cells was particularly evident for glutamine synthetase, with heavy labeling restricted to the outer retina adjacent to photoreceptor cell bodies (ONL) in RPC-treated eyes (Figure 8D–G). Caspase, a marker of apoptosis, was diminished in RPC-treated retinas and elevated in shams (Figure 8H,I), along with regional alterations in cytochrome oxidase expression suggesting changing metabolic activity in response to altered function demands.

## 3. Discussion

Here we show that intravitreal transplantation of unmodified retinal progenitor cells results in photoreceptor preservation and amelioration of functional deficits associated with a rod–cone dystrophy, as assessed by multiple measures. The RPC-based treatment effect was first demonstrated by transplanting allogeneic rat cells and then replicated using the analogous human cells, paving the way for translation of this approach to the clinic. The transplanted cells were well tolerated in the vitreous cavity, with or without systemic immune suppression (for xenogeneic and allogeneic models, respectively), and survived for a prolonged period, either as free-floating clusters in the vitreous cavity or adhering non-invasively to surrounding intraocular structures.

These results differ from previous studies in that immature retinal cells were used to implement a neuroprotective strategy rather than replace host photoreceptor cells. Consequently, a relatively simple intravitreal placement was sufficient to achieve therapeutic efficacy. This is notable for a number of reasons. First, most prior work in the RCS rat has focused on subretinal implantation of cells, particularly RPE cells, with the goal of local cell replacement. Subretinal injection, however, is itself associated with a notable sham effect in the RCS rat [24]. The use of sham controls is therefore critical in this model [25], but false positives due to the sham effect remain a concern when interpreting rescue data following any subretinal intervention. In contrast, the sham effect associated with intravitreal injection is marginal in comparison. Furthermore, intravitreal injection is less technically demanding than subretinal placement, thereby facilitating clinical implementation of this approach.

Intravitreal RPC grafts remained avascular, and no angiogenic response was observed in the host eyes. RPCs were well tolerated as allografts, whereas immune suppression was required for sustained survival of human-to-rat xenografts, despite concerns this might diminish functional responses in RCS eyes [22]. We have observed previously that introduction of RPCs induces a host microglial/macrophage response, that this reaction is graft-directed but relatively mild, and that it subsides over time and is not associated with disruption of host retinal architecture [26]. Here we confirm that the RPC treatment effect was evident in both allograft and xenograft models, regardless of immune modulation. The retinas of host animals showed preservation of not only the outer nuclear layer (ONL) but also the adjacent outer plexiform layer (OPL), where photoreceptor cells synapse onto second-order neurons. Preservation of the OPL appears to provide a relatively early anatomic marker of efficacy. An early functional indication of efficacy was relative preservation of ERG response in RPC-treated animals. RPCs are endogenous to the retina and thus might be expected to exhibit an enhanced safety profile over ectopic cell types, particularly those associated with epithelial–mesenchymal transition and myofibroblastic transformation. Cells assuming such contractile properties are associated with retinal traction, retinal detachment, and proliferative vitreoretinopathy (PVR). In contrast, intravitreal RPCs were not observed to exert tractional forces on the retina in this study.

The photoreceptor rescue associated with non-integrated, intravitreal RPCs is consistent with a diffusible trophic effect, and in fact, the cells elaborate a range of factors with potential neurotrophic activity. Basic fibroblast growth factor (bFGF) is known to have trophic effects in the RCS retina [27]. Osteopontin (OPN) has previously been linked to macrophage chemotaxis [28], as well as the retinal ganglion cell [29] and photoreceptor rescue [30], in addition to its non-ocular role as a bone-associated extracellular protein. Midkine family member pleiotrophin is known to be expressed by neural cells [31], as is midkine itself, and the latter has been associated with photoreceptor rescue [32]. Additional factors expressed by hRPCs include MANF [33], PEDF [34], and humanin [35].

In addition to preservation of photoreceptor cells, including features of the outer segment and synaptic layers, response to RPC treatment was also seen in the non-neuronal cells of the retina. Such responses included relative normalization of morphology and gene expression levels, as seen with connexin 43 in RPE and GFAP in Mueller cells and astrocytes.

Viewed as a delivery system, a cell-based approach has the potential for sustained release, as well as the potential for modulated expression of multiple factors based on interaction with the diseased tissue [2]. From a clinical standpoint, intravitreal delivery lends itself to re-dosing of patients in a way that subretinal delivery does not.

Taken together, the advantages exhibited by this combination of cell type and delivery method serve to facilitate clinical administration. The proof-of-principle data, together with formal toxicology studies and the clinical protocol, contributed to opening an IND with the FDA, and initial clinical trials in late-stage retinitis pigmentosa (RP) have been undertaken (NCT02320812; NCT03073733). It will therefore soon be possible to evaluate findings from the animal studies presented here in light of data obtained from patients with RP.

## 4. Materials and Methods

### 4.1. Cell Culture

Rat RPCs (R28) were purchased from Kerafast (Boston, MA, USA), cultured in DMEM (Sigma-Aldrich, St. Louis, MO, USA) supplemented with 10% FBS, 1% MEM non-essential amino acids, 1% MEM vitamins, and 1% GlutaMax-I (Gibco, Gaithersburg, MD, USA), as described in the product sheet.

Human RPCs were originally derived from fetal tissue and cryopreserved at low passage number. Cells were later thawed as needed and cultured on fibronectin-coated flasks in Advanced DMEM/F12 supplied with 1x GlutaMax-I CTS, 1% N2 supplement CTS, 20 ng/mL EGF, and 20 ng/mL FGF-basic CTS (Gibco, Gaithersburg, MD, USA) for 2 days for all experiments with medium changed 1 day after thawing. Medium was collected 2 days after cells thawed (1 day after medium changed), with the exact hours of growing and the number of cells in the flask recorded for secreted protein amount calculation in ELISA assays.

Cells used for transplantation were harvested with TrypLE (Gibco, Gaithersburg, MD, USA) and formulated in BSS PLUS Irrigating solution (Alcon Laboratories, Fort Worth, TX, USA) to the desired concentration right before transplantation. Cell number and viability were re-assessed after completing surgery. The cell number was consistently within 20% of the final dosing concentration, and the viability was >85% after transplantation.

Approval for use of human cells was obtained from the human stem cell research oversight (hSCRO) committee, and the proposed use was reviewed by the Institutional Review Board (IRB).

### 4.2. RNA Extraction

Total RNA was extracted from cells or retinas and processed using either an RNeasy Mini Kit or an AllPrep DNA/RNA/Protein Mini Kit (Qiagen, Valencia, CA, USA) following the manufacturer’s instructions. DNase I was added in process to eliminate traces of genomic DNA. RNA was quantified via spectrophotometer (ND-1000; NanoDrop Technologies Inc., Wilmington, DE, USA) for the optical density (OD) absorption ratio OD260 nm/OD280 nm 2.00–2.10, OD260 nm/OD230 nm 2.00–2.20.

### 4.3. Microarray Analysis

Results from microarray chips were normalized using the sketch-quantile method (Expression Console™ ver.1.1 software, Affymetrix). Microarray analysis was performed in JMP Genomics (SAS Americas, Cary, NC, USA) using a one-way ANOVA, and statistical significance was established by setting the False Discovery rate threshold to α < 0.05 [36]. Principal component analysis (PCA) plots were generated in JMP Genomics as part of the analysis. Individual gene expression changes were considered functionally significant for fold change >|±2-fold| and α < 0.05.

### 4.4. Real-Time Polymerase Chain Reaction Assay

Two micrograms of total RNA was reverse transcribed with an Omniscript RT kit (Qiagen, Valencia, CA, USA) and 10 µM random primers (Sigma-Aldrich, St. Louis, MO, USA) according to the manufacturer’s instructions. Real-time PCR was performed using a ViiA 7 Real-Time PCR System (Applied Biosystems, Foster City, CA, USA). Also, 2x TaqMan Gene Expression Master Mix and TaqMan^®^ Gene Expression Assays (Applied Biosystems, Foster City, CA, USA) were used for qPCR reaction, and each reaction was performed in triplicate from a single biological replicate. All reactions involving human cells were repeated multiple times to ensure consistency of results). Graphs were plotted, and analysis was performed with the ΔΔCt method (QuantStudio™ Real-Time PCR Software and DataAssist 3.01, Applied Biosystems) or JMP Genomic software version 5.1 (SAS Americas, Cary, NC, USA). All data points are expressed as mean ± standard deviation (SD). TaqMan Gene Expression assays used in this study are listed in Table 1.

### 4.5. Immunocytochemistry

Cells were plated and grown on four-well chamber slides for 48 h, then fixed for 20 min in 4% paraformaldehyde and washed three times with DPBS, followed by permeabilizing and blocking with blocking buffer (0.3% Triton X-100 and 5% donkey serum) for 1 h at room temperature, followed by another DPBS wash. Primary antibodies were prepared in antibody buffer (0.3% Triton X-100 and 1% donkey serum) and incubated with cells overnight at 4 °C. After washing with DPBS, cells were incubated with anti-mouse Alex Fluor 546 secondary antibody (Thermo Fisher Scientific, Carlsbad, CA, USA) for 1 h at room temperature in the dark, followed by several DPBS washings. The chamber slides were mounted with VECTASHIELD antifade mounting medium with DAPI (Vector laboratories, Burlingame, CA, USA), and images were acquired with a Nikon Ti microscope, NIS-elements viewer, and analyzed by Image J software. ImageJ is a free Java-based image processing platform developed by the National Institutes of Health (https://www.nih.gov/, accessed on 23 May 2017). Antibody controls were processed identically, except that incubation with primary antibody was eliminated. Primary antibodies used in this study are Adenovirus type 2 E1A (M73, Abcam, Cambridge, MA, USA), Ki-67 (B56), Nestin (25/NESTIN), Sox2 (030-678) (BD Biosciences, San Jose, CA, USA), Vimentin (V9, Sigma-Aldrich, St. Louis, MO, USA), and GD2 (14.G2a, Millipore, Temecula, CA, USA). The percentage of positive profiles was calculated by counting those profiles expressing specific immunoreactivity, divided by the cells identified by DAPI staining in 10–11 randomly selected fields.

### 4.6. FACS

hRPCs were trypsinized to single-cell suspension 48 h after thawing, with medium changed 24 h after thawing. For Sox2, Ki67, Nestin, and GFAP staining, cells were fixed with fixation buffer and permeabilized with PERM Buffer III (BD Biosciences, San Jose, CA, USA) before staining. Following centrifugation and supernatant removal, antibodies were added to the cells and incubated in the dark for 30–60 min. After several washes, cells were analyzed on a BD FACSAria II flow cytometer and the data plotted using FlowJo software (FlowJo, LLC, Ashland, OR, USA). Unstained and isotype-stained controls were included in all samples. The antibodies used were anti-Nestin (25/NESTIN); anti-Sox2 (030-678); anti-Ki-67 (B56); anti-GFAP (1B4); anti-Human HLA-ABC (G46-2.6); an anti-Human HLA-DR, DP, DQ (Tu39); and their corresponding isotype controls (BD Biosciences, San Jose, CA, USA).

### 4.7. ELISA

Media collected 2 days after hRPC thawing (1 day after medium changed) was used to measure hRPC OPN and FGF-basic secretion. OPN and FGF-basic levels were measured by the Human Osteopontin OPN Quantikine ELISA kit and Human FGF basic Quantikine ELISA kit (R&D systems, Minneapolis, MN, USA), following the manufacturer’s instructions. OD450 and OD540 were detected on a BioTek Synergy HT microplate reader (Winooski, VT, USA). To create a standard curve, CurveExpert software version 1.40 (Hyams Development) was used to generate a best fit curve through the standard points. All samples were performed in triplicate.

### 4.8. Animals

Dystrophic Royal College of Surgeons (RCS) rats (rdy+ p+) were used for this study. All animals were pigmented with the brown-eyed, dark-hooded RCS phenotype. The rats were bred in a colony at the University of California, Irvine, and maintained under a 12 h light/dark cycle (maximum 7.7 lux at cage level) and fed a Teklad irradiated standard diet, Harlan #292. Animals were housed and handled in adherence with guidelines set forth by the Institutional Animal Care and Use Committee (IACUC) at the University of California, Irvine. To mimic the baseline conditions under which rats are evaluated following treatment with xenografted cell therapeutics, selected litters of RCS rats received daily dexamethasone injections (1.6 mg/kg i.p.) for a period of 2 weeks starting at the age of weaning (P21) and were also maintained on cyclosporine-A (Bedford Labs, Bedford, MA, USA) administered in the drinking water (210 mg/L) from weaning age until the time of euthanasia. All procedures were carried out in accordance with the ARVO Statement for the Use of Animals in Ophthalmic and Vision Research.

### 4.9. Transplantation

Animals were anesthetized via IP injection of ketamine (30 mg/kg) and dexmedetomidine (0.1 mg/kg). A 2 µL aqueous solution of either vehicle (balanced salt solution: BSS PLUS) or cell suspension containing a low/medium/high/extra-high dose of hRPC was injected into the vitreous cavity at age P21–P22. After surgery, atipamezole (1.0 mg/kg) was given IP. Animals were returned to the cage with soft food.

### 4.10. Optomotor Response (OR) Threshold

Visual acuity was measured based on spatial frequency discrimination, tested at P45, P60, and P90 using an OptoMotry testing apparatus (Cerebral Mechanics). Using the testing protocol established by Douglas, et al. [37], rats were placed inside the OptoMotry apparatus, and their response was measure for both the clockwise and counterclockwise directions. Acuity was quantified by increasing the spatial frequency of the grating using a staircase progression until the reflexive head movements ceased, thereby obtaining a maximum threshold.

### 4.11. Electroretinography (ERG)

Animals were evaluated by full-field electroretinography (ERG) using a Ganzfeld stimulator at three different time points: P45, P60, and P90. All animals were dark adapted overnight (>12 h), and all testing was done under a dim red light. Before the test, each animal’s eye was dilated with 1 drop each of topical Tropicamide 1% ophthalmic solution (Bausch & Lomb) and Phenylephrine 2.5% ophthalmic solution (Akorn), and animals were then anesthetized by intraperitoneal injection using a combination of Ketamine 70 mg/kg (Mylan Institutional Galway) and Xylazine 3.5 mg/kg (Akorn or equivalent). Animals were placed on a heated platform (37 °C) to maintain a constant body temperature during the ERG test. ERGs were recorded from both eyes simultaneously using gold wire loops placed on each cornea, with a drop of methylcellulose applied to the corneal surface. A stainless steel needle electrode (Rhythmlink, Columbia, SC, USA) was placed subdermally at the base of the tail as the ground, and a stainless steel needle electrode was placed subdermally in the ventral midline of the chin as the reference. Measurements were performed using an Espion e3 recording unit coupled to the ColorDome Ganzfeld LED stimulator (Diagnosys LLC, Lowell, MA, USA). The protocol included scotopic flash light intensities of 0.5 and 5 cds/m^2^, a photopic flash light intensity of 50 cds/m^2^ after 10 min of light adaptation, and a 30 Hz photopic flicker at an intensity of 25 cds/m^2^ (background of 30 cds/m^2^).

### 4.12. Histology

The terminal endpoint was age P90–P100, i.e., Day 69–79 post injection. Rats were humanely euthanized by CO_2_ asphyxiation. Eyes were enucleated and fixed either in Davidson’s solution for a paraffin embedding process or in 0.1 M cacodylate buffered 4% paraformaldehyde for a cryo-embedding process for 48 h at 4 °C, then embedded either in paraffin (Polysciences, Warrington, PA, USA) or in O.C.T. Compound (Fisher, Hampton, NH, USA), respectively. For each eye, sagittal sections of 5 μm thickness (paraffin-embedded samples) or 10 μm thickness (O.C.T.-embedded samples) were cut from the nasal-to-lateral side of the glove, and every 5th slides were stained with hematoxylin and eosin (H&E). All stained slides were examined under a Nikon SMZ25 stereomicroscope (Nikon, Tokyo, Japan), and selected slides from the peri-optic nerve area were imaged using a Nikon Eclipse Ti-inverted research microscope (Nikon, Japan) for morphological evaluation of the retinal architecture and outer nuclear layer (ONL) thickness.

### 4.13. IHC

For hRPC-treated eyes, human-specific marker (STEM121) was used to evaluate donor cell survival and engraftment. Specific markers including the neural progenitor marker nestin, proliferation markers Ki-67 or PNCA, neural lineage markers such as DCX or MAP2 or recoverin or opsin, and glial lineage marker GFAP can be used for evaluation of the cell fate of surviving donor cells. The markers Isolectin B4 and Iba1 were used to evaluate the host immune response to the grafted cells.

### 4.14. Statistics

ERG and OR results were analyzed in JMP (SAS Americas, Cary, NC, USA) using a Student’s *t*-test.

Microarray analysis was performed in JMP Genomics (SAS Americas, Cary, NC, USA) using a one-way ANOVA and setting the False Discovery rate to α < 0.05 [36].

## Figures and Tables

**Table 1:** TaqMan Gene Expression Assay used in real-time PCR.

Gene	Assay ID
Human GAPDH	Hs99999905_m1
Human NANOG	Hs02387400_g1
Human Nestin	Hs00707120_s1
Human RHO	Hs00892431_m1
Human SOX2	Hs01053049_s1
Human SPP1	Hs00959010_m1
Human TERT	Hs00972656_m1
Rat CNTF	Rn00755092_m1
Rat bFGF	Rn00570809_m1
Rat GAPDH	Rn01775763_g1
Rat GDNF	Rn01402432_m1
Rat GFAP	Rn01253033_m1
Rat JAG1	Rn00569647_m1
Rat KDR	Rn00564986_m1
Rat MDK	Rn00675549_g1
Rat Ki67	Rn01451446_m1
Rat NANOG	Rn01462825_m1
Rat Nestin	Rn01455599-g1
Rat PDGFc	Rn00579958_m1
Rat PTN	Rn00567035_m1
Rat Rho	Rn00583728_m1
Rat RT1-CE1	Rn04222416_gH
Rat RT1-CE16	Rn04222422_gH
Rat RT1-Db1	Rn01429350_m1
Rat RT1-M3-1	Rn00575896_g1
Rat PEDF	Rn00709999_m1
Rat Sox2	Rn01286286_g1
Rat SPP1	Rn00681031_m1
Rat TGFb1	Rn00572010_m1
Rat VEGFa	Rn01511602_m1

## Data Availability

Data are contained within the article.
